# Comparison of anterior column reconstruction techniques after en bloc spondylectomy: a finite element study

**DOI:** 10.1038/s41598-023-45736-6

**Published:** 2023-10-31

**Authors:** Agoston Jakab Pokorni, Mate Turbucz, Rita Maria Kiss, Peter Endre Eltes, Aron Lazary

**Affiliations:** 1 In Silico Biomechanics Laboratory, National Center for Spinal Disorders, Királyhágó St. 1-3, Budapest, 1126 Hungary; 2 School of PhD Studies, Semmelweis University, Budapest, Hungary; 3 Department of Mechatronics, Optics and Mechanical Engineering Informatics, Faculty of Mechanical Engineering, Budapest University of Technology and Economics, Műegyetem Rkp. 3., Budapest, 1111 Hungary; 4 Department of Spine Surgery, Department of Orthopaedics, Semmelweis University, Budapest, Hungary

**Keywords:** Biomedical engineering, Computational science, Medical research

## Abstract

Total en bloc spondylectomy (TES) effectively treats spinal tumors. The surgery requires a vertebral body replacement (VBR), for which several solutions were developed, whereas the biomechanical differences between these devices still need to be completely understood. This study aimed to compare a femur graft, a polyetheretherketone implant (PEEK-IMP-C), a titan mesh cage (MESH-C), and a polymethylmethacrylate replacement (PMMA-C) using a finite element model of the lumbar spine after a TES of L3. Several biomechanical parameters (rotational stiffness, segmental range of motion (ROM), and von Mises stress) were assessed to compare the VBRs. All models provided adequate initial stability by increasing the rotational stiffness and decreasing the ROM between L2 and L4. The PMMA-C had the highest stiffness for flexion–extension, lateral bending, and axial rotation (215%, 216%, and 170% of intact model), and it had the lowest segmental ROM in the instrumented segment (0.2°, 0.5°, and 0.7°, respectively). Maximum endplate stress was similar for PMMA-C and PEEK-IMP-C but lower for both compared to MESH-C across all loading directions. These results suggest that PMMA-C had similar or better primary spinal stability than other VBRs, which may be related to the larger contact surface and the potential to adapt to the patient’s anatomy.

## Introduction

Primary spine tumors only account for 11% of primary musculoskeletal tumors^1^, but the spine is the most common site for skeletal metastasis, representing 39% of all bone metastases^2^. Since the first description of the total en bloc spondylectomy (TES) and the vertebral body replacement (VBR) by Szava in 1959^3,4^, TES has been found to be an effective treatment for particular cases of primary and metastatic spine tumors^5^.

Following TES, both anterior reconstruction and posterior instrumentation are required^6^. The main complications of anterior support include subsidence of the device into the adjacent vertebra, dislocation of the VBR, and fracture or collapse of the anterior implant^7^. Various solutions have been applied to replace vertebrae after TES and restore biomechanical stability. Anterior reconstruction can be achieved with autologous or allogeneic bone graft, individually formed polymethylmethacrylate (PMMA), or manufactured implants such as titanium mesh cage (TMC) and expandable cage (EC) mainly made from metal alloys or polymers^7,8^.

Several previous studies have investigated the biomechanical properties and clinical outcomes of different VBRs after corpectomy or TES, but only a few have compared more than two types of VBR under identical conditions, and mostly retrospective clinical studies have included PMMA as well^8–17^.

Finite element (FE) analyses allow the measurement of parameters that are difficult to assess by other methods^18^; therefore, FE can provide valuable insight into the biomechanical effects of vertebral replacement devices^19^. Lacroix et al.^19^ and Wang et al.^20^ investigated different bone grafts after single-level corpectomy in the lumbar spine. Lacroix et al.^19^ found that using a femoral bone graft to replace a single vertebra is better in terms of load transfer than using tibial or fibular grafts, but the position of a bone graft can have a significant influence on the resulting stresses. Similarly, Wang et al.^20^ found that subsidence can be reduced by proper positioning of the bone graft and that the surface configuration of the contact between the VBR and bony endplate has the most effect on endplate stress distribution. The same conclusion was reached by Xu et al.^21^ regarding the matching of the endplate and the implant. They compared a perfectly fitting 3D printed VBR with a well-fitting and mismatched TMC and found that a smaller contact surface caused by the mismatch will produce higher endplate stresses and lower construct stability. In contrast, the FE analyses of Zander et al.^22^ showed that, as many parameters affect the contact pressures on the endplate, a large VBR is not always advantageous from a mechanical point of view. However, in a study by Chen et al. that compares a TMC, a titanium EC, and a composite EC, the authors conclude that although all three devices are similarly effective in restoring spinal stability, the larger contact area of the composite device helps to dissipate stresses and thus reduce peak stresses^23^, which are considered to be a significant factor in cage subsidence^24,25^.

The present study aimed to compare the initial stability of a femur graft (FEM-GRAFT-C), a polyetheretherketone implant (PEEK-IMP-C), a titan mesh cage (MESH-C), and a custom made PMMA replacement (PMMA-C) using a finite element model of the lumbar spine after a TES of the third lumbar (L3) vertebra. These replacements are used commonly in our national center for spinal disorders and were selected to include representative implant and reconstruction types frequently utilized in clinical practice. The four different VBRs were compared based on their rotational stiffness, segmental range of motion (ROM), and the maximum and distribution of von Mises stress on the adjacent bony endplates. To the best of the authors’ knowledge, this is the first paper that compares the PMMA with other VBRs used after total en bloc spondylectomy in an in silico investigation.

## Methods

### Construction of the finite element model of the intact lumbar spine

**Figure 1:**
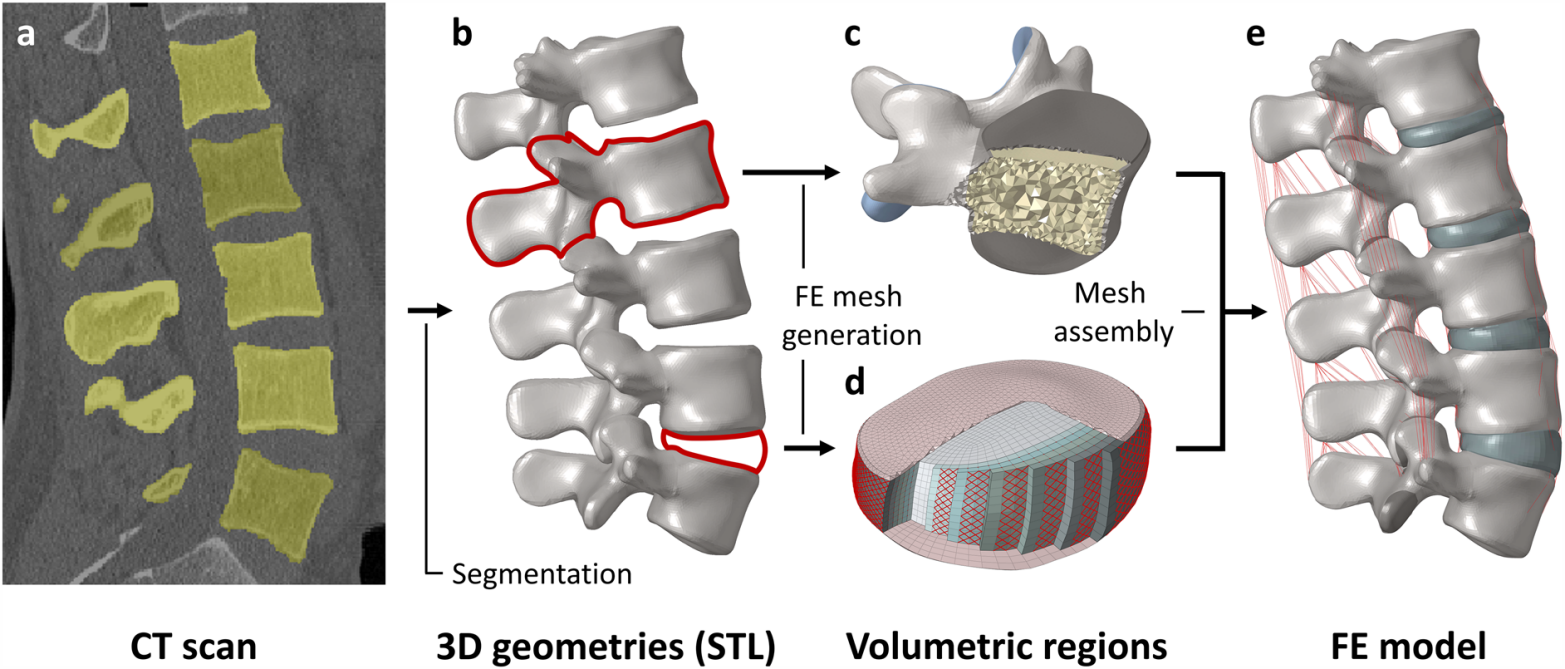
Construction of the finite element model of the intact lumbar spine. The 3D geometries of the vertebrae were created by segmentation of the QCT images (**a**–**b**). The volumetric regions of the vertebrae (**c**) and the intervertebral discs (**d**) were defined in accordance with the data published in the literature^27^. The finite element model of the lumbar spine was created by assembling the meshes of the vertebrae and intervertebral discs and adding the ligaments (**e**).

The construction and validation of the lumbar spine finite element model were detailed in a previous article^26^, so only a summary of the modelling process is given here (Fig. 1). A geometric model of the lumbar spine was created by segmenting a quantitative computed tomography (QCT) scan of a 24-year-old man in Mimics software (Mimics Research, Mimics Innovation Suite v23.0, Materialise, Leuven, Belgium). The vertebrae consisted of the 1 mm thick cortical shell, the trabecular bone, the posterior elements, and the 0.5 mm thick bony endplates made in 3-Matic software (Mimics Research, Mimics Innovation Suite v21.0, Materialise, Leuven, Belgium) as surface meshes. The intervertebral discs consisted of the nucleus pulposus, the annulus fibrosus ground substance and fibers, as well as the 0.5 mm thick cartilaginous endplate. The facet joints were modeled as 0.25 mm thick layers of cartilage with an initial spacing of 0.5 mm and frictionless contact between adjacent surfaces.

The seven major ligaments of the lumbar spine (Anterior Longitudinal Ligament, Posterior Longitudinal Ligament, Ligamentum Flavum, Intertransverse Ligament, Supraspinous Ligament, Interspinous Ligament, Facet Capsular Ligament) were modeled as nonlinear uniaxial spring elements. The volume meshes were created and the final model was compiled in HyperWorks software (Altair Engineering, Inc., Troy, Michigan, United States). Table 1 summarizes the material properties used in the modelling process. The finite element models were validated with in vivo, in vitro, and in silico data. Validation was performed for various pure and combined loads based on the ROM, intervertebral rotation (IVR), intradiscal pressure (IDP), and facet joint force (FJF)^26^. The reliability of the validation was increased by using several relevant validation parameters^27,28^, and we have applied three different loading conditions to enhance the quality of the model's prediction, similar to a comparative study among eight acknowledged finite element models of the lumbar spine^27^. Comparison with available data from the literature showed that the models have realistic motion (for both the ROM and the IVR) against the applied loads^27,29–34^. Correlation with the in vivo data of Wilson et al. confirmed that the models give reasonable FJF values^35^. The IDP results compared to the in vitro measurements from the literature indicated excellent reliability of the models^36,37^. The finite element simulations were solved in Abaqus Standard (v2021, Dassault Systemes, Vélizy-Villacoublay, France).

**Table 1:** Summary of the material properties and element types.

Material	Element type	Constitutive law	Material properties
Cortical bone	C3D4	Linear elastic	E = 10,000 [MPa], ν = 0.3^22^
Trabecular bone	C3D4	Linear elastic	E = 100 [MPa], ν = 0.2^38^
Posterior elements	C3D4	Linear elastic	E = 3500 [MPa], ν = 0.25^38^
Bony endplate	C3D4	Linear elastic	E = 1200 [MPa], ν = 0.29^39^
Cartilaginous endplate	C3D4, C3D5	Linear elastic	E = 23.8 [MPa], ν = 0.42^40^
Nucleus pulposus	C3D8H	Mooney-Rivlin	C10 = 0.12; C01 = 0.03^41^
Annulus fibrosus ground substance	C3D8H	Mooney-Rivlin	C10 = 0.18; C01 = 0.045^42^
Annulus fibrosus fibers	T3D2	Nonlinear stress–strain curves^38,42,43^	
Ligaments	SPRINGA	Nonlinear stress–strain curves^42^	
Facet cartilage	C3D6	Neo-Hooke	C10 = 5.36; D1 = 0.04^40^
Titanium screws, rods, and pins	C3D4	Linear elastic	E = 110,000 [MPa], ν = 0.3^19,39^
Trabecular bone graft	C3D4	Linear elastic	E = 100 [MPa], ν = 0.2^38^
Femur allograft	C3D4	Linear elastic	E = 16,700 [MPa], ν = 0.3^44^
Titanium mesh	C3D4	Linear elastic	E = 35,000 [MPa], ν = 0.3^45,46^
PEEK	C3D4	Linear elastic	E = 3600 [MPa], ν = 0.3^39^
PMMA	C3D4	Linear elastic	E = 3000 [MPa], ν = 0.4^37^

### Vertebral body replacement construct and surgical finite element model development

**Figure 2:**
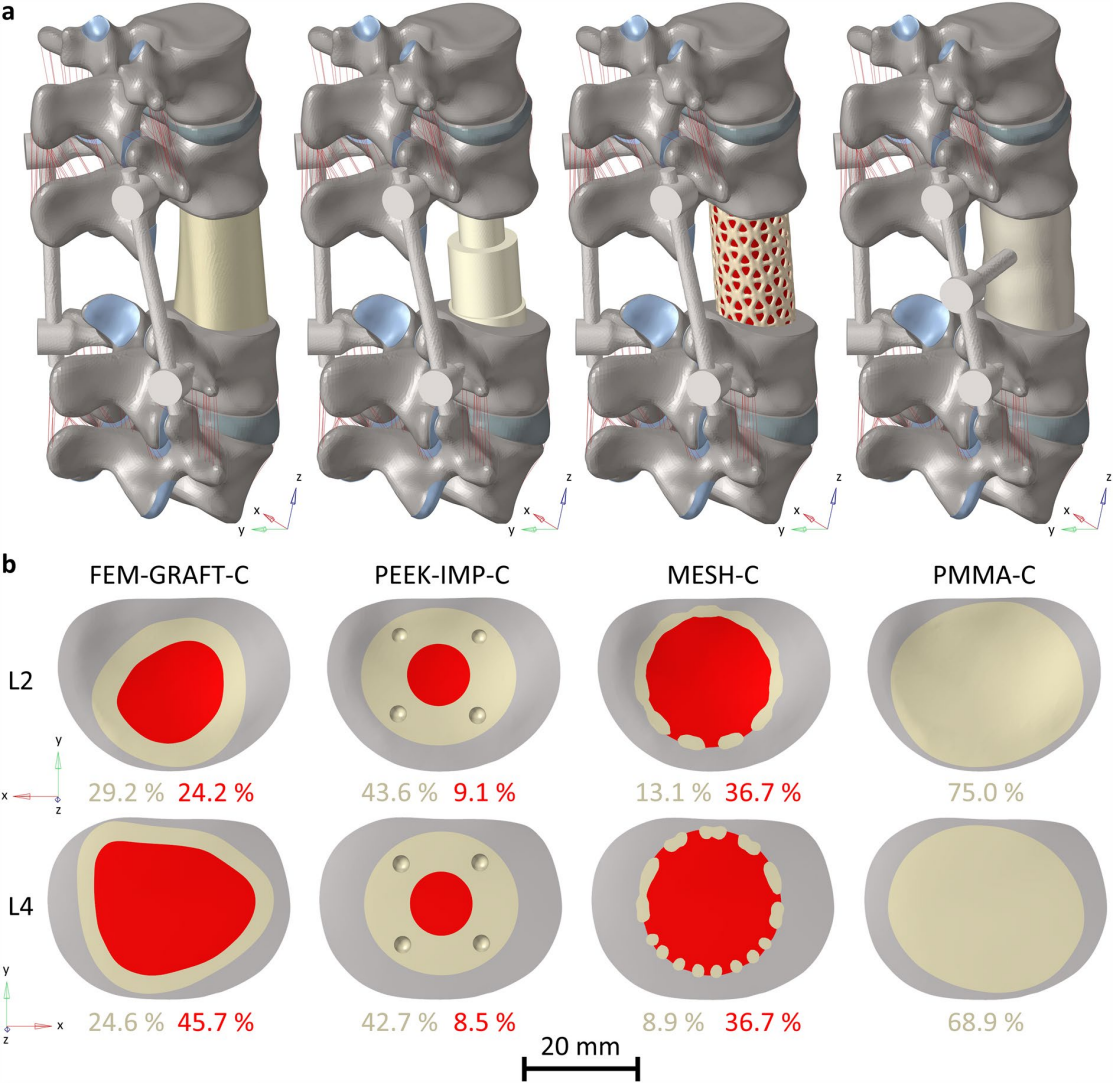
The surgical finite element models of the lumbar spine with the four investigated vertebral body replacements (**a**), and the contact surface of the VBRs (yellow) and the bone grafts (red) at the two adjacent endplates in percentage of the endplate area (**b**).

The bony and soft tissues were identical for each lumbar model after surgery as the variable parameter was the type of vertebral replacement. Total en bloc spondylectomy was modelled with complete removal of the L3 vertebra, the adjacent discs and cartilaginous endplates, as well as the ligaments connecting the second and fourth lumbar vertebrae through the third. The L3 vertebra was chosen to be removed to allow a healthy segment both above and below the posterior fixation. A titanium posterior fixation system, consisting of pedicle screws (Ø6 mm × 45 mm) and rods (Ø 5.5 mm) connected the second and the fourth lumbar vertebra, and four different VBRs were inserted to replace the removed vertebra (Fig. 2a). The geometries of a femur allograft (FEM-GRAFT-C), a titanium mesh cage (MESH-C), and a PEEK expandable implant cage with titanium pins (PEEK-IMP-C) were created by segmenting computed tomography (CT) scans similarly to the vertebrae in Mimics. The volume bounded by the vertebrae and the resulting geometries of the replacements were filled with bone graft. The dimensional parameters of the MESH-C are summarized in Supplementary Fig. S1. The geometry of the PMMA cage (PMMA-C) was formed from a cylindrical body based on an ellipse with diameters 29 and 34 mm in Fusion 360 (Autodesk, Sab Francisco, California, United States). Figure 2b summarizes the contact surfaces of the VBRs and the bone grafts on the bony endplate of the adjacent vertebrae. Based on the surgeon’s recommendation and institutional experience, an extra screw was inserted in the PMMA-C at the level of the removed L3's right pedicle, connecting the replacement to the posterior rod on the right side. The screws had a fixed connection to the PMMA-C and the vertebrae^19,46,47^. The meshing and assembling of the surgical model were completed in Hyperworks. There was friction contact between the replacements and the vertebra with a friction coefficient of 0.8^23^. The lower endplate of the fifth lumbar vertebra was fixed, while a follower load of 400 N and a torque of 7.5 Nm were applied through the upper endplate of the first lumbar vertebra^23^ to simulate the upper body weight and the effect of muscle forces in flexion, extension, left and right lateral bending, and left and right axial rotation. The rotational stiffness of the construct, segmental ROM, the maximum von Mises stress in the bony endplate of the second lumbar (L2) and fourth lumbar (L4) vertebra, and in the posterior rods, and the distribution of the von Mises stress in the two bony endplates were evaluated in Abaqus Standard.

### Ethical approval

Ethical Approval was given by the National Ethics Committee of Hungary and the National Institute of Pharmacy and Nutrition (reference number: OGYÉI/163-4/2019). All methods were carried out in accordance with relevant guidelines and regulations. Informed consent was obtained from the participant.

## Results

**Figure 3:**
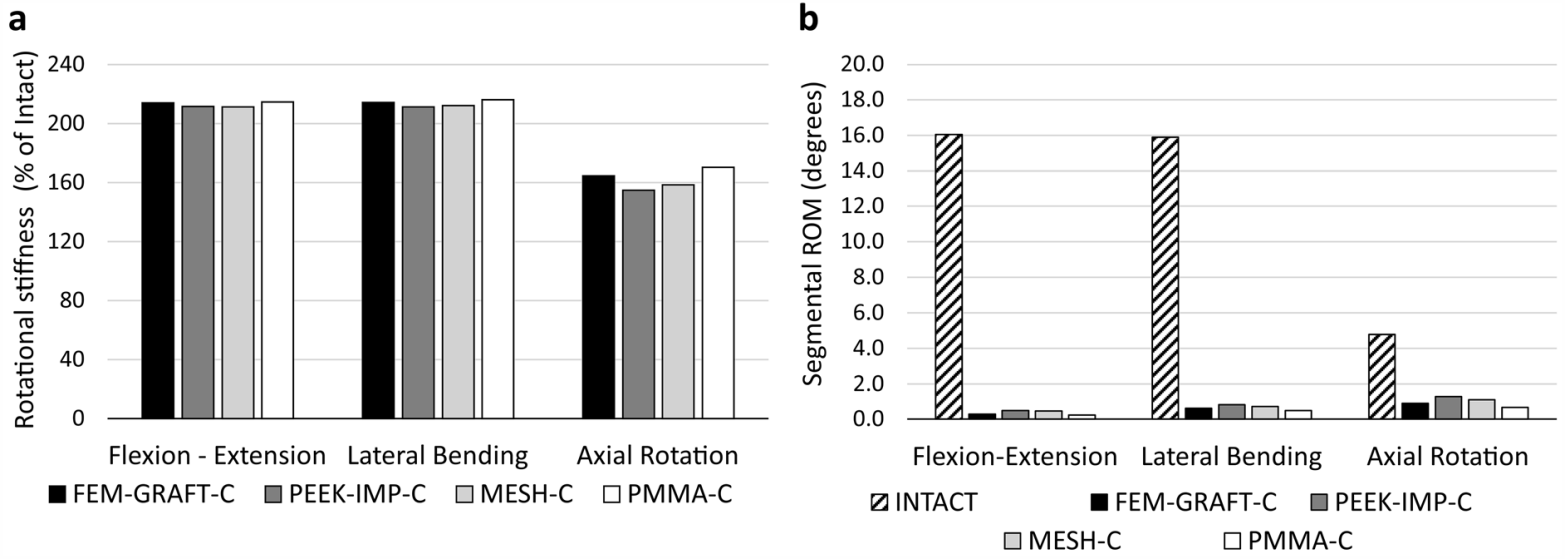
Kinematic parameters of the surgical model of the lumbar spine. The rotational stiffness of the models in % of the intact model (**a**). Segmental ROMs of the fixed L2–4 segment in flexion–extension, lateral bending, and axial rotation (**b**). ROM: range of motion.

**Figure 4:**
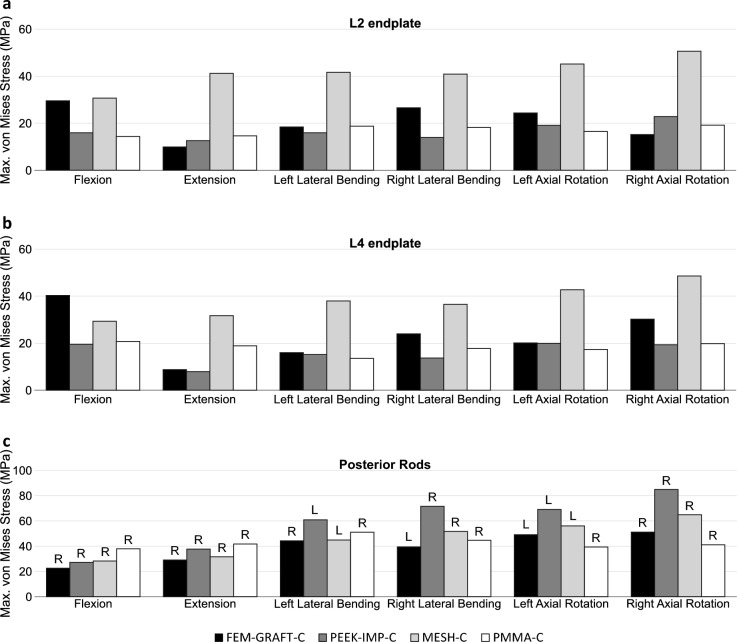
Maximum von Mises stress in different load cases in the inferior bony endplate of L2 (**a**), in the superior bony endplate of L4 (**b**), and in the posterior rods (**c**). L: left rod, R: right rod.

**Figure 5:**
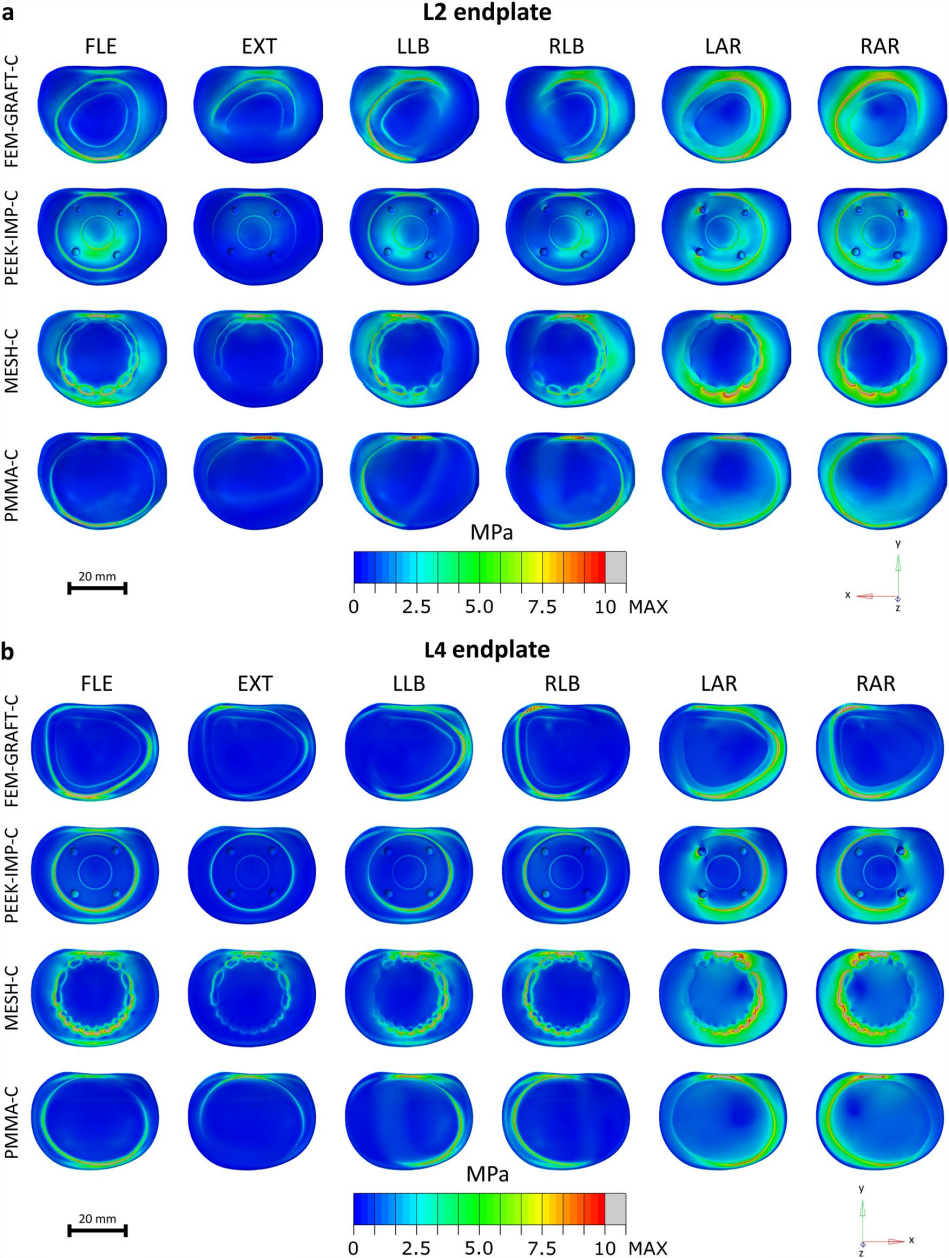
Distribution of von Mises stress in different load cases in the inferior bony endplate of L2 from a bottom view (**a**) and in the superior bony endplate of L4 from a top view (**b**). *FLE* flexion, *EXT* extension, *LLB* left lateral bending, *RLB* right lateral bending, *LAR* left axial rotation, *RAR* right axial rotation.

The present study assessed the rotational stiffness of the surgical model (Fig. 3a), the ROM of the fixed segments (Fig. 3b, Supplementary Table S1), the maximum von Mises stresses in the bony endplates of the L2 and L4 vertebra and in the posterior rods (Fig. 4, Supplementary Table S2), and distributions of the von Mises stress in the bony endplates of the adjacent vertebrae (Fig. 5).

The rotational stiffness of the constructs measured under the same load showed that the values were notably increased compared to the intact specimen in all loading directions (Fig. 3a), but also that the four anterior reconstructions yielded similar values in flexion–extension (median: 213%, minimum: 211%, maximum: 215%), lateral bending (median: 213%, minimum: 211%, maximum: 216%) and axial rotation as well (median: 161%, minimum: 155%, maximum: 170%). Although the PMMA-C had the highest stiffness for all loading directions (215%, 216%, and 170% for flexion–extension, lateral bending, and axial rotation, respectively), the MESH-C had the lowest rotational stiffness in flexion–extension (211%), while the PEEK-IMP-C had the lowest stiffness in lateral bending (211%) and axial rotation (155%).

The ROM of the fixed segments showed that the reconstructions have seriously reduced the motions of these segments in flexion–extension (median: 0.4°, which is 2% of Intact) and lateral bending (median: 0.7°, which is 4% of Intact), while in axial rotation the motions have been reduced only to a lesser extent (median: 1°, which is 21% of Intact) (Fig. 3b, Supplementary Table S1). For both flexion–extension, lateral bending and axial rotation, the PEEK-IMP-C had the largest (0.5°, 0.8°, and 1.3°, respectively) and the PMMA-C had the smallest (0.2°,0.5°, and 0.7°, respectively) movements. For the same loads, the second largest movement occurred for the MESH-C (0.5°, 0.7°, and 1.1°, respectively) and the second smallest for the FEM-GRAFT-C (0.3°, 0.6°, and 0.9°, respectively). Consequently, the difference between the largest and smallest ROMs was 2% in flexion–extension, 2% in lateral bending, and 12% in axial rotation, compared to the ROM of the intact L2–4 segment.

The von Mises stresses measured in the bony endplates adjacent to the VBRs for different load cases showed that both the maximum stress and the stress distribution differed greatly between the different reconstructions (Figs. 4a,b, 5 and Supplementary Table S2). In the inferior endplate of L2, the highest maximum stress occurred in the MESH-C in all load cases and was 30.7, 41.2, 41.7, 40.9, 45.2, and 50.6 MPa in flexion, extension, left lateral bending, right lateral bending, left axial rotation and right axial rotation, respectively (Fig. 4a). The lowest maximum stress in the inferior endplate of L2 occurred in the FEM-GRAFT-C in extension and right axial rotation (9.9 and 15.2 MPa), in the PEEK-IMP-C in left and right lateral bending (15.9 and 13.9 MPa), and in the PMMA-C in flexion and left axial rotation (14.4 and 16.5 MPa). In the superior endplate of L4, the highest maximum stress occurred in the FEM-GRAFT-C in flexion and was 40.3 MPa, and it occurred in the MESH-C in extension, left lateral bending, right lateral bending, left axial rotation and right axial rotation, and it was 31.7, 38.0, 36.5, 42.7, 48.6 MPa, respectively (Fig. 4b). The lowest maximum stress in the superior endplate of L4 occurred in the PEEK-IMP-C in flexion, extension, right lateral bending, and right axial rotation (19.5,7.9,13.7, and 19.3 MPa, respectively), and in the PMMA-C in left lateral bending and left axial rotation (13.5 and 17.4 MPa).

The maximum von Mises stresses in the posterior rods indicated that, in addition to load on the adjacent endplates, the load on the rods is also different for the various replacements (Fig. 4c and Supplementary Table S2). The highest maximum stress in the posterior rods occurred in the PMMA-C in flexion and extension (37.9 and 41.7 MPa), and in the PEEK-IMP-C in left and right lateral bending (60.9 and 71.6 MPa), and in left and right axial rotation (69.1 and 84.9 MPa). The lowest maximum stress in the posterior rods occurred in the FEM-GRAFT-C in flexion, extension, left lateral bending, and right lateral bending (22.6, 29.1, 44.2, and 39.4 MPa, respectively), and in PMMA-C in left and right axial rotation (39.4 and 41.1 MPa). The largest difference between the maximum stresses for different VBRs was measured in right axial rotation between PEEK-IMP-C and PMMA-C (43.9 MPa), while the smallest difference was observed in extension between PMMA-C and FEM-GRAFT-C (12.7 MPa).

High stresses occurred mainly at the outer edge of the contact surface between the VBRs and the endplates for both adjacent vertebrae (Fig. 5). Although, in the PEEK-IMP-C, high stresses also occurred at the inner edge of the replacement in flexion (Fig. 5a) and around the pins in axial rotation (Fig. 5a,b). The posterior part of the endplates was typically highly stressed in all load cases, although the FEM-GRAFT-C and the PMMA-C in flexion had lower stresses in this area. In lateral bending and axial rotation, the FEM-GRAFT-C, the PEEK-IMP-C, and the MESH-C also experienced higher stresses in the lateral areas of the contact surface between the replacement and the endplate, while in the PMMA-C, similar to flexion and extension, the front or posterior areas of the contact surfaces were more highly stressed.

## Discussion

Several solutions are currently available for the anterior reconstruction of the spine after total en bloc spondylectomy. Four different types of VBR were compared in the present study after L3 TES using finite element models of the lumbar spine. The stability and biomechanics of the surgical constructs were analyzed based on the rotational stiffness, segmental ROM, the maximum von Mises stresses in the bony endplates of the L2 and L4 vertebra and in the posterior rods, and distribution of the von Mises stress in the bony endplates adjacent to the replacements.

The stability of the structures was determined by their rotational stiffness, similar to Wang et al.^48^. The rotational stiffness increased notably in all three loading directions compared to the intact model for all models. However, it increased least in axial rotation, consistently with previous in vitro and in silico results^11,21,48,49^. There was no significant difference in stiffness between the individual replacements, meaning that all devices provide similar adequate construct stability, although, PMMA-C demonstrated the greatest stiffness in all directions. The lack of difference was in agreement with the study by Pflugmacher et al.^11^, who found that the type of VBR had only a small effect on stiffness in anterior reconstructions combined with posterior fixation. Shannon et al.^13^ found, similarly to us, that an anterior PMMA replacement with posterior fixation provides greater stiffness than an intact spine and therefore provides sufficient stability. Although the stiffness of the construct is crucial for initial fixation and long-term stability^50^, recent research suggests that excessive stiffness can cause the degeneration of adjacent segments^51,52^.

The segmental ROMs of the instrumented segments were considerably decreased for all devices in all loading directions, ensuring good primary stability for all VBRs. The reduction was greatest in flexion–extension and lateral bending, with a median of 2% and 4%, respectively, and the smallest in axial rotation, with a median of 21% of the intact ROM. This smaller reduction presumably does not affect the stability of the devices since the median rotation here did not exceed 1° either, and the ROM was relatively less reduced in this case due to the small displacements of the intact spine in axial rotation. However, the in silico results of Rohlmann et al.^53^ showed that the rotations measured in axial rotation might even increase slightly with the increase of the elastic modulus of the bone graft, in contrast to the values measured in flexion and extension. This change suggests axial rotational stability may need increased attention in the clinical evaluation of devices with short posterior fixation and added anterior bone graft. Ulmar et al.^54^ tested 12 human spinal specimens after single-level corpectomy of L2 reconstructed with an expandable cage and an antero-lateral plate, posterior fixation, or both. They concluded that only combined antero-posterior instrumentation has sufficient axial rotational stability. Wang et al.^48^ investigated a new type of one-level VBR, and found that artificial pedicles increase the rotational stability of the device. This could be the reason for the better rotational stability of PMMA-C, since the screw connecting the anterior replacement to the posterior fixation acts like an artificial pedicle, not only preventing the dislocation of the device but also increasing rotational stability. In axial rotation, the differences between the VBRs were also larger, as the difference between the largest and smallest ROMs was greater than 12%, whereas, in flexion–extension and lateral bending, it was well below 3%. A similar trend was observed by Chen et al.^23^ in the lumbar spine and the sacrum, as the largest difference between the devices in the axial rotation was 8.4%, while in flexion–extension and lateral bending, the difference was only 4.6% and 4.8%, respectively. Similar results were obtained by Knop et al.^10^ as well, who found that for VBRs with posterior fixation, the difference between devices was 71.4% of the intact ROM in the axial rotation, while it was only 28% and 5.6% in flexion–extension and bending, respectively.

Cage subsidence is a common complication of TES^55–59^. It can be observed as early as one month after surgery^57^, and can be associated with poor clinical outcomes^59^. In vitro studies showed that besides bone mineral density and the endplate condition, the stress concentrations on the interface between the VBR and the endplate significantly impact subsidence^24,60,61^. Based on the maximal von Mises stresses in the bony endplates of the adjacent vertebrae, our results showed, that the MESH-C has most likely the greatest risk of subsidence as this replacement had the highest peak stress in all load cases in both endplates, except for the flexion in the L4 endplate. The high risk of subsidence of titanium mesh cages has also been reported by previous clinical studies^55–59^, although it has been particularly highlighted mainly in multilevel fixation^57–59^, and the risk of subsidence might be reduced by proper positioning and size^20,58,60,62^, In our study, the maximum stresses of the PEEK-IMP-C and the PMMA-C were similar apart from extension in the L4 endplate, and the maximum stress was significantly, in most load cases 50%, less than that of the MESH-C for both replacements, suggesting that they have similar effects on the bony endplates and are less subjected to subsidence than MESH-C. In addition to the difference in the elastic moduli of the devices, this substantial difference between the maximum stresses is also expected to be strongly influenced by the size of the contact surface between the endplates and the replacements since PMMA-C, PEEK-IMP-C, and FEM-GRAFT-C account for 72%, 43%, and 27% of the endplate area on average, respectively, whereas MESH-C accounts for only 11%. The combination of high maximum endplate stress and small contact surface is in accordance with the results of previous studies that have found that the size of the VBR influences the subsidence and that the replacements with a large footprint have more resistance to subsidence^21,25,47,60,61^. Chen et al.^23^ found that the hydroxyapatite/polyamide VBR has significantly lower maximum stresses on the endplate compared to the titanium mesh cage in all load cases, which may be partly due to the enlarged contact surface of this device, as this helps to disperse the stress. Similarly, a retrospective study by Zhang et al.^63^ showed that the incidence of cage subsidence was significantly lower with hydroxyapatite/polyamide replacements than with titanium mesh cages. Like Fang et al.^16^, Rajpal et al.^17^ found that both PMMA and bone implants had a much lower incidence of subsidence than metal implants, although the rate of revision surgery was highest in the case of bone implants. Over the past few years, there has been growing interest in the use of 3D printing for creating custom implants as vertebral replacement, as they could match the patient’s unique anatomy, ensuring optimal fit^64^. These implants show promising results in preventing subsidence^65–67^, although the procedure is still developing, and it’s important to conduct long-term follow-ups to ensure it’s safe and effective^64^.The stability of a VBR depends on the stress distributions, as well as on the maximum stresses, since the end plate is not uniformly resistant to subsidence. The PMMA-C mainly loaded the posterior, anterior, and lateral areas of the bony endplates for both adjacent endplates, which may have contributed to its stability, as these are the strongest areas of the bony endplates^60,68^. A similar distribution was obtained in the L4 endplate in the FEM-GRAFT-C, while in the L2 endplate and in the case of MESH-C and PEEK-IMP-C, the central areas were more heavily loaded on the edges of the implants. The more advantageous stress distribution may be responsible for the lower incidence of subsidence observed in PMMA and bone replacements^16,17^. PMMA is individually formed^7^, so it does not need to be cut to size like the bone graft or the titanium mesh cage^8^. This allows the replacement to fit perfectly to the endplate and achieve a good stress distribution by loading the suitable areas^69^. Previous studies have found that VBRs with a better fit to the bony endplate have increased compressive strength^24^ and reduced peak stresses due to better stress distributions^20,21,70^, resulting in a reduced incidence of subsidence. However, in addition to the advantageous properties of PMMA, its disadvantages should also be considered, including thermal injury^71^, dislodgment^69^, and the lack of bony fusion^13^. Nevertheless, PMMA is significantly cheaper than other solutions with similar clinical results to expandable cages^15,17^.

The maximum von Mises stress results in the posterior rod highlight that the model is not fully symmetric due to the patient-specific geometry, and therefore the maximum stress is mostly in the right rod, even in otherwise symmetrical loads such as flexion and extension. In contrast to other replacements, in the case of the PMMA-C, the maximum stress consistently occurs in the right rod, possibly due to the influence of the additional screw, although the maximum stress values remain relatively close to those observed with the other VBRs. Nonetheless, monitoring stresses within the rods remain crucial for the long-term stability of the surgical treatment, as overloading of the posterior fixation can potentially lead to complications^7,72^. However, the results indicate that for all the investigated replacements and load cases, the maximum stress of the posterior rods remained well below the yield strength and even the fatigue strength of titanium alloy^73^. For the reconstructions without connection between the posterior fixation and the anterior replacement (FEM-GRAFT-C, PEEK-IMP-C, MESH-C), the maximum stresses in the rods occurred in axial rotation considering all loading directions, similar to Wu et al.^74^. Conversely, for the PMMA-C, the highest maximum von Mises stresses occurred in lateral bending, and the lowest maximum stresses occurred in axial rotation, similar to Wang et al.^48^ since the extra screw presumably acts as an artificial pedicle.

This is the first in silico study to compare PMMA replacement to other vertebral body replacements, however the study has some limitations due to the nature of the finite element method. Simplifications have been applied to the implants, and the misfit of the VBRs has not been investigated. The study aimed to investigate the primary stability of the replacements; therefore the effects of bony fusion and thermal injury were not considered. The comparison of different VBRs based on finite element simulation is intended to show trends and relations rather than the presentation of the actual data. Subsequently, further research is needed to investigate the effects of multi-level surgery, the long-term stability of the devices with fusion bone, and the impact of age-related osteoporosis on the stability of the devices.

## Conclusion

A finite element comparison of four vertebral body replacement techniques after total en bloc spondylectomy is represented in this paper. All four replacements provided a good level of stability based on the instrumented segment’s rotational stiffness and ROMs. However, there was a smaller motion reduction in axial rotation, and the difference between the devices was also the largest in this load case. The posterior rod stresses showed that the maximum stress increases slightly with PMMA-C in flexion and extension but decreases significantly in axial rotation. Moreover, the endplate stresses suggest that MESH-C has most likely the greatest risk of subsidence, while PMMA—firstly studied in this surgical scenario—has the lowest. These findings align with previous studies that concluded that PMMA provides clinically adequate results. The contact surface area of the VBR device with the endplate influences the chance of subsidence, and the device which fits on a patient-specific endplate geometry could provide better stability and favorable stress distribution.

To the best of the authors’ knowledge, this is the first paper that compares the PMMA with other VBRs used after total en bloc spondylectomy in an in silico investigation.

### Supplementary Information

Supplementary Information.

## Data Availability

The authors believe that the “Methods” section and Supplementary cover the investigated topics satisfactorily. Correspondence and requests for materials should be addressed to P.E.E.
