# Supplementary material for: Comparison of anterior column reconstruction techniques after en bloc spondylectomy: a finite element study
Source: Sci Rep. 2023 Oct 31;13:18767. doi: 10.1038/s41598-023-45736-6 (PMC10618450; doi:10.1038/s41598-023-45736-6)
Supplement: Supplementary file 1 — Supplementary Information. [file 41598_2023_45736_MOESM1_ESM.pdf]

Comparison of anterior column reconstruction techniques after en bloc spondylectomy: a finite element study

Agoston Jakab Pokorni[1,2], Mate Turbucz[1,2], Rita Maria Kiss[3], Peter Endre Eltes[1,4*] and Aron Lazary[1,4*]

[1] In Silico Biomechanics Laboratory, National Center for Spinal Disorders, Budapest, Hungary
[2] School of PhD Studies, Semmelweis University, Budapest, Hungary
[3] Department of Mechatronics, Optics and Mechanical Engineering Informatics, Faculty of Mechanical Engineering, Budapest University of Technology and Economics, Budapest, Hungary
[4] Department of Spine Surgery, Department of Orthopaedics, Semmelweis University, Budapest, Hungary

[*] The authors equally contributed to the manuscript

Supplementary Materials

Model	Segment	Segmental ROM (deg (% of Intact))		
		Flexion-Extension	Lateral Bending	Axial Rotation
INTACT	L1-2	6.92 (100.0)	6.23 (100.0)	2.30 (100.0)
	L2-4	16.05 (100.0)	15.88 (100.0)	4.80 (100.0)
	L4-5	6.79 (100.0)	6.67 (100.0)	3.08 (100.0)
FEM-GRAFT-C	L1-2	6.88 (99.4)	6.17 (99.0)	2.25 (97.8)
	L2-4	0.30 (1.8)	0.63 (3.9)	0.89 (18.5)
	L4-5	6.72 (99.0)	6.63 (99.4)	3.05 (99.1)
PMMA-C	L1-2	6.90 (99.8)	6.19 (99.3)	2.25 (97.6)
	L2-4	0.23 (1.4)	0.49 (3.1)	0.68 (14.2)
	L4-5	6.73 (99.1)	6.64 (99.5)	3.05 (99.1)
MESH-C	L1-2	6.90 (99.7)	6.19 (99.3)	2.26 (98.0)
	L2-4	0.46 (2.8)	0.73 (4.6)	1.11 (23.1)
	L4-5	6.73 (99.0)	6.65 (99.7)	3.06 (99.5)
PEEK-IMP-C	L1-2	6.87 (99.3)	6.17 (99.0)	2.25 (97.8)
	L2-4	0.50 (3.1)	0.83 (5.2)	1.28 (26.6)
	L4-5	6.70 (98.7)	6.62 (99.3)	3.05 (99.2)

Supplementary Table S1. Segmental ROMs of the different models in different loading directions. ROM: range of motion.

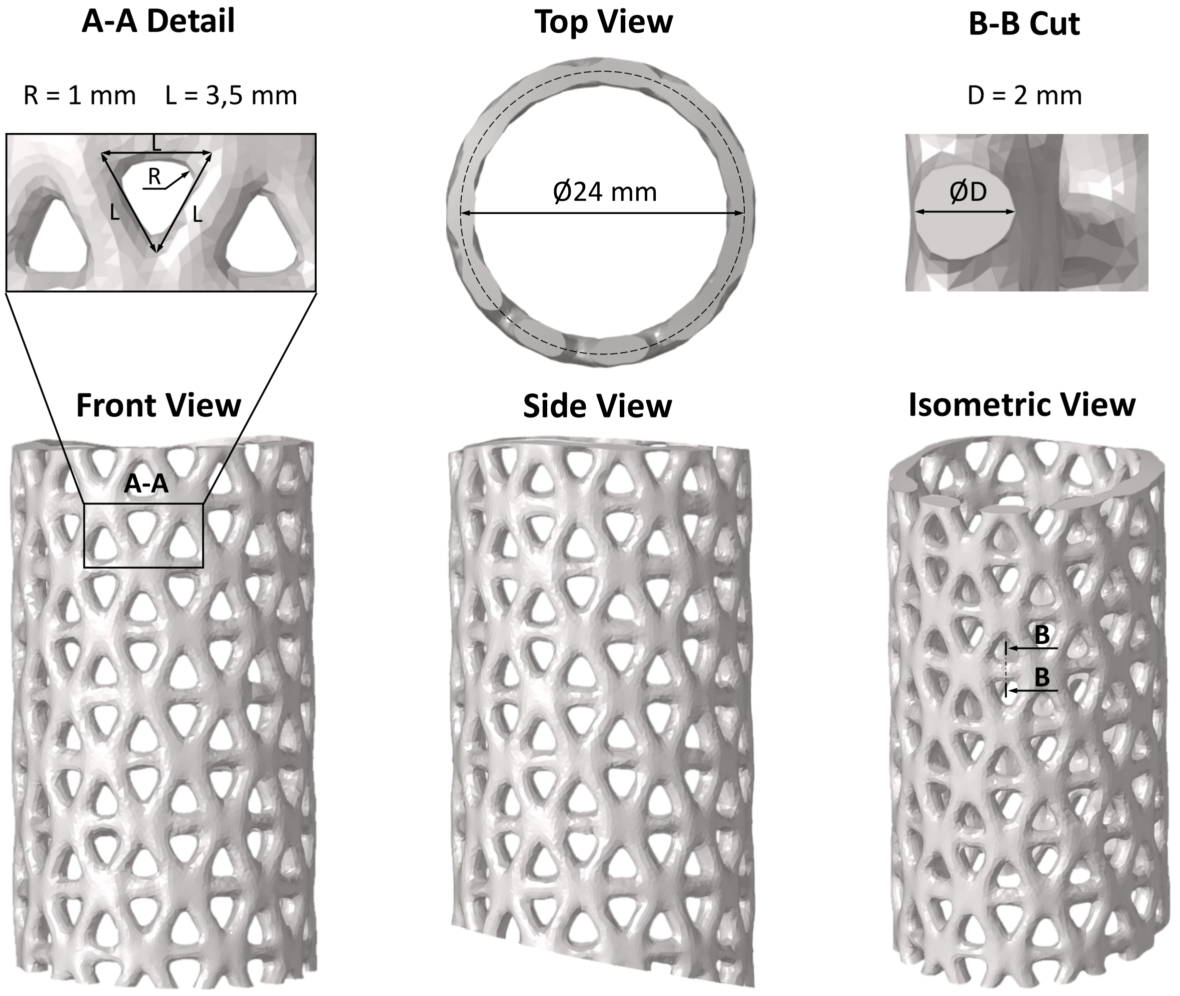

Supplementary Figure S1. The dimensional parameters of the titanium mesh.

Model	Part	Maximum von Mises Stress (MPa)					
		Flexion	Extension	Left Lateral Bending	Right Lateral Bending	Left Axial Rotation	Right Axial Rotation
FEM-GRAFT-C	Inferior Bony Endplate of L2	29.6	9.9	18.4	26.6	24.4	15.2
	Superior Bony Endplate of L4	40.3	8.7	16.0	24.0	20.1	30.2
	Left Screw in L2	21.8	29.0	34.5	27.2	45.6	51.6
	Right Screw in L2	30.5	26.4	31.8	32.4	41.6	38.6
	Left Screw in L4	25.1	37.2	38.7	32.9	48.6	47.4
	Right Screw in L4	32.8	45.4	32.4	32.5	48.2	52.6
	Left Rod	18.0	20.2	31.7	39.4	49.0	48.6
	Right Rod	22.6	29.1	44.2	34.0	48.7	51.1
PEEK-IMP-C	Inferior Bony Endplate of L2	15.9	12.6	15.9	13.9	19.1	22.8
	Superior Bony Endplate of L4	19.5	7.9	15.2	13.7	19.9	19.3
	Left Screw in L2	37.4	24.7	35.3	42.5	71.1	59.6
	Right Screw in L2	41.5	27.1	43.3	40.1	48.6	60.4
	Left Screw in L4	36.5	40.8	48.0	45.8	62.2	63.4
	Right Screw in L4	41.6	52.0	46.4	50.9	67.6	67.0
	Left Rod	18.8	31.7	60.9	34.8	69.1	68.9
	Right Rod	27.2	37.7	45.8	71.6	65.3	84.9
MESH-C	Inferior Bony Endplate of L2	30.7	41.2	41.7	40.9	45.2	50.6
	Superior Bony Endplate of L4	29.3	31.7	38.0	36.5	42.7	48.6
	Left Screw in L2	36.1	17.9	26.4	39.3	58.6	51.6
	Right Screw in L2	42.4	17.9	45.2	29.1	44.9	48.6
	Left Screw in L4	34.9	42.3	45.0	42.2	55.5	52.3
	Right Screw in L4	39.0	54.7	42.2	48.2	58.1	58.0
	Left Rod	22.5	23.5	44.9	36.9	56.0	53.1
	Right Rod	28.3	31.6	42.3	51.7	55.3	64.9
PMMA-C	Inferior Bony Endplate of L2	14.4	14.6	18.8	18.2	16.5	19.2
	Superior Bony Endplate of L4	20.7	18.9	13.5	17.7	17.4	19.8
	Left Screw in L2	28.8	17.5	24.5	31.1	36.7	39.4
	Right Screw in L2	33.1	27.2	32.6	30.4	34.9	35.1
	Screw in PMMA	10.9	27.4	16.8	28.2	25.9	28.6
	Left Screw in L4	26.3	38.5	39.5	33.3	37.5	36.9
	Right Screw in L4	24.9	36.9	24.1	21.9	38.6	50.7
	Left Rod	19.4	18.5	30.7	32.9	39.0	37.0
	Right Rod	37.9	41.7	51.1	44.6	39.4	41.1

Supplementary Table S2. Maximum von Mises Stress in different parts, models, and loading directions.
